# Data from three prospective longitudinal human cohorts of prenatal marijuana exposure and offspring outcomes from the fetal period through young adulthood

**DOI:** 10.1016/j.dib.2016.10.005

**Published:** 2016-10-18

**Authors:** Gabrielle L. McLemore, Kimberlei A. Richardson

**Affiliations:** a Morgan State University, Department of Biology, SCMNS, 1700 East Cold Spring Lane, Baltimore, MD 21251, USA; b Howard University College of Medicine, Department of Pharmacology, 520 W Street, NW Suite 3408, Washington, DC 20059, USA

**Keywords:** Prospective longitudinal human cohorts, Prenatal marijuana exposure (PME), Offspring outcomes, Fetal period, Young adulthood, Ottawa Prenatal Prospective Study (OPPS), Maternal Health Practices and Child Development Study (MHPCD), The Generation R Study (Gen R), Cognitive skills, Psychological skills, Behavioral skills

## Abstract

This article includes data from three prospective longitudinal human cohorts of prenatal marijuana exposure (PME) and offspring outcomes from the fetal period through young adulthood. The table herein contains an overview of the major adverse effects associated with PME from the following human cohorts: (1) The Ottawa Prenatal Prospective Study (OPPS); (2) The Maternal Health Practices and Child Development Study (MHPCD); and (3) The Generation R Study (Gen R). In the OPPS, fetal gestational age was measured and age-appropriate standardized neuropsychological instruments were used to assess neonatal responses, and infant–child and adolescent–young adult cognitive and behavioral skills. In the MHPCD, birth length and weight, neonatal body length, and infant–child sleep, cognition, and behavioral parameters were measured. In the Gen R, birth weight and growth were measured, as were infant–child attention and aggression. The data in this article are in support of our report entitled “Prenatal Cannabis Exposure - The "First Hit" to the Endocannabinoid System” (K.A. Richardson, A.K. Hester, G.L. McLemore, 2016) [13].

**Specifications Table**

**Table:** 

Subject area	*Biology*
More specific subject area	*Prenatal marijuana exposure and the first hit to the endogenous cannabinoid system**[13]*
Type of data	*Table*
How data was acquired	*Prospective longitudinal human cohort data derived from the OPPS, MHPCD, and Gen R investigations with follow-up assessments of offspring from the fetal period through young adulthood*
Data format	*Formatted*
Experimental factors	*Prenatal marijuana exposure and offspring outcomes from the fetal period through young adulthood*
Experimental features	*The OPPS assessed the effects of prenatal tobacco, alcohol, and marijuana exposure in low-risk, middle-class, Caucasian Canadian women. The MHPCD assessed the effects of prenatal alcohol and marijuana exposure in Caucasian and African American women of low socioeconomic status. The Gen R assessed the effects of prenatal marijuana exposure in a multi-ethnic cohort of women in Rotterdam, The Netherlands.*
Data source location	*N/A*
Data accessibility	*Data is within this article.*

**Value of the data**

• This dataset is of value to the scientific community because it can serve as a reference for other researchers interested in the adverse effects of prenatal marijuana use on offspring outcomes from the fetal period through young adulthood.
• These data are of value to the scientific community because they can open doors for new collaborative research efforts between physician-scientists (neonatologists), psychiatrists, drug addiction counselors, and/or basic drug abuse and addiction research scientists.
• These data highlight the need for more extensive basic, clinical, and translational research on the impact of prenatal marijuana exposure on the development of cognitive and psychological processes in exposed offspring.
• These data highlight the need for researchers to develop novel strategies for improving the health of and healthcare for pregnant women and their children.

## 1 Data

The data described herein are adapted from three articles [1], [10], [15]. This table highlights the cognitive, psychological, and behavioral effects of prenatal marijuana exposure with follow-up assessments of offspring outcomes from the fetal period through young adulthood (Table 1).

## 2 Experimental design, materials, and methods

The OPPS assessed the effects of prenatal tobacco, alcohol, and marijuana exposure in a low-risk, middle-class, mostly Caucasian, and predominantly Canadian cohort of women. The offspring of participants were followed until the age of 18–22 years [6], [7], [8].

The MHPCD assessed the effects of prenatal alcohol and marijuana exposure in a cohort of Caucasian (43%) and African American (57%) women of low socioeconomic status who were predominantly single (71%) in Pittsburgh, Pennsylvania, USA. Follow-up assessment data of offspring have been reported up to age 14 [2], [3].

The Gen R is an ongoing, population-based, large-scaled, multi-ethnic prospective cohort investigation of prenatal marijuana exposed offspring from the fetal period to early childhood in Rotterdam, The Netherlands. [4], [5], [9], [11], [12].

## Figures and Tables

**Table 1:** Three human prospective longitudinal cohorts of prenatal marijuana exposed offspring from the fetal period through young adulthood.

**Study**	**Fetus**	**Neonate**	**Infant–Child**	**Adolescence–Young adult**
**Ottawa Prenatal Prospective Study (OPPS)** [6], [7], [8]	↓ Gestational age	↓ Response to light ↑ Startle response ↑ Tremors	**3 years:** ↑ Motor skills **4 years:** ↓ Memory ↓ Verbal scores **6 years:** ↓ Attention ↑ Impulsivity ↑ Hyperactivity	**9-12 years:** ↓ Visual perception ↑ Impulsivity **13-16 years:** ↓ Concentration ↓ Visual memory ↓ Verbal reasoning **18-22 years:** ↓ Response inhibition **Response inhibition as measured via fMRI:** ↓ Cerebellum activity ↑ Bilateral PFC activity ↑ Premotor cortexactivity **Working memory as measured via fMRI:** ↓ Medial PFC activity ↓ Dorsolateral PFC activity ↓ Ventrolateral PFC activity ↑ Left medial PFC activity ↑ Inferior frontal gyrus activity ↑ Left cerebellum activity
**Maternal Health Practices and Child Development Study (MHPCD)** [2], [3]	↓ Birth length *(after 1*^*st*^ *trimester exposure only)* ↑ Birth weight *(after 3*^*rd*^ *trimester exposure)*	↓ Body length **Subpopulation:** *slight* changes in EEG traces	**9 months:** ↓ Mental development ↓ BSID scores **3 years:** ↓ Short-term memory ↓ Verbal reasoning *(African Americans only)* **3 years:** ↓ Sleep efficiency ↑ Nocturnal arousals ↑ Wake-time after sleep onset **6 years:** ↓ Concentration ↓ Overall IQ score ↓ Verbal reasoning ↓ Quantitative reasoning ↓ Short-term memory ↑ Impulsivity ↑ Hyperactivity ↑ Delinquency	**10 years:** ↓ Abstract reasoning ↓ Visual reasoning ↓ Concentration ↓ Internalization (Implosion) ↓Learning and memory ↓ IQ score ↑ Externalization (Explosion) ↑ Depression ↑ Impulsivity ↑ Hyperactivity ↑ Delinquency **14 years:** ↑ Delinquency **16 years:** *slight* ↓ in fine motor coordination *slight* ↑ in visual-motor coordination
**Subsample of the Generation R Study, the Generation R Focus Study**[5,^9^,^11^,12]	↓ Birth weight ↑ Growth (from 2^nd^ trimester to parturition)		**18 months:** ↓ Attention ↑ Aggression (for girls only)	

Adapted from Calvigioni et al. [1]**,** Huizink [10]**,** and Wu et al. [15].

↑ - Increased; ↓ - Decreased; BSID - Bayley Scales of Infant Development; fMRI - Functional Magnetic Resonance Imaging; Response inhibition - an indicator of executive control, refers to one’s ability to suppress inappropriate actions or impulses, which undergird goal-oriented and adaptable responses to dynamic surroundings [14].
